# Ventral tegmental area dopaminergic circuits participates in stress-induced chronic postsurgical pain in male mice

**DOI:** 10.1186/s12868-023-00842-z

**Published:** 2024-01-09

**Authors:** Weizhen Liu, Wang Wang, Ziliang Wang, Ying Xing

**Affiliations:** 1 Department of Physiology and Neurobiology, School of Basic Medical Sciences, Zhengzhou University, Zhengzhou, 450001 Henan China; 2 The Academy of Medical Sciences of Zhengzhou University, Zhengzhou, 450001 Henan China

**Keywords:** Chronic postsurgical pain, Stress, Dopamine, Ventral tegmental area, Optogenetics, Chemogenetics

## Abstract

**Background:**

Chronic postsurgical pain (CPP) markedly impairs patients’ quality of life. Research has shown that chronic stress may extend incisional nociception in male mice. Dopaminergic (DAergic) neurons in the ventral tegmental area (VTA) are integral to stress-related mental disorders (including major depressive disorder, anxiety disorders, and PTSD) and pain. However, the impact of chronic social defeat stress (CSDS) on mesolimbic dopamine (DA) transmission in the development of CPP is yet to be established. It remains uncertain whether the dopamine signals in the rostral anterior cingulate cortex (rACC), which regulate pain, derive from the VTA. This study aims to explore the role of VTA-rACC dopaminergic circuits in a mouse model of CPP induced by CSDS.

**Methods:**

We conducted CSDS on C57BL/6 J wild-type male mice (n = 12–16 mice/group) and DAT-cre male mice (n = 10–12 mice/group). After 10 days of CSDS, a left posterior plantar incision was made to establish a mouse model of CPP. Paw withdrawal thresholds (PWTs) were evaluated using Von-Frey fibre stimulation. The open field test (OFT) and elevated plus maze test (EPM) were used to assess pain-related negative emotions. We used immunofluorescence staining and Western Blot to analyse D1, D2, c-Fos, and TH expression. DAergic fibre projections in the VTA-rACC neural pathway were traced using retrograde tracing and immunofluorescence staining. Optogenetics and Chemogenetics were employed to manipulate DAergic neurons in the VTA and their axons in the rACC.

**Results:**

The ipsilateral PWTs in male C57BL/6 J mice significantly decreased after surgery, returning to baseline after seven days. Conversely, in CSDS mice, ipsilateral PWTs remained reduced for at least 30 days post-incision. A significant reduction in TH-positive neurons expressing c-Fos in the VTA of CPP mice was observed 15 days post-incision. Activating DAergic neurons significantly improved ipsilateral PWTs and locomotor performance in the OFT and EPM in CPP mice post-incision. Additionally, D1 expression in the rACC was found to decrease in CPP mice, and this reduction counteracted the increase in PWTs caused by activating DAergic neuron axon terminals in the rACC.

**Conclusion:**

CSDS results in chronicity of postsurgical nociception and anxiety-like negative emotions, with alterations in DA transmission playing a role in CPP. Specific activation of DAergic neurons mitigates nociceptive responses and anxiety-like bahaviors, possibly mediated by D1 receptors in the rACC.

**Supplementary Information:**

The online version contains supplementary material available at 10.1186/s12868-023-00842-z.

## Introduction

Chronic postsurgical pain (CPP) markedly affects patients’ quality of life after surgery [1]. However, the clinical efficacy of drug therapy is often limited [2]. Approximately 5–10% of patients who experience postoperative pain develop into severe chronic pain, with the incidence increasing annually [3]. There is growing attention to the impact of psychosocial factors on pain. Research suggests that negative emotions, such as anxiety and depression, in perioperative patients significantly affect the chronic progression of postsurgical pain [4, 5]. Understanding the role of negative emotions in the perception and modulation of chronic postsurgical pain will provide a theoretical basis for drug therapy.

Psychological stress-induced negative emotions effectively mirror the psychological state of preoperative patients. Exposure to chronic stress, either before or after surgery could prolong postsurgical pain [6, 7]. Common chronic stresses, such as restraint, sleep deprivation, chronic forced swimming, and maternal separation, are typically artificial rather than social stress in rodents [8–14]. Chronic social defeat stress (CSDS) in mice has been demonstrated to replicate the psychosocial stress experienced by humans and is extensively used to study behavioural changes due to chronic stress [15, 16]. Wang et al. emphasize that the CSDS model is an ethologically valid rodent model, reflecting long-term physiological and behavioral phenotypes similar to human depression and anxiety, and it accurately depicts individual stress response variations [17]. Jian Lu et al. provided insights from rodent studies on the transmission of social defeat stress, offering valuable perspectives for understanding human social stress dynamics [18].

Evidence reveals that prolonged stress adversely affects the dopamine (DA) reward system in the mesolimbic system, leading to anhedonia [19, 20]. Increasing research supports that dopaminergic (DAergic) neurons in the ventral tegmental area (VTA) and their downstream pathways are integral to pain perception and modulation [21–28]. Activation of DAergic neurons in the VTA produces acute antinociceptive effects [29]. Thus, the influence of DA reward system dysfunction on the onset and persistence of pain under stress requires further exploration.

Neuroimaging studies have demonstrated that both physiological and psychological pain trigger neuronal activation in the anterior cingulate cortex (ACC), a region involved in pain perception and modulation [30–32]. Research showes that ACC ablation heightens nociceptive responses, while its activation could suppress spinal dorsal horn neuron activity, yielding analgesic effects [33]. Recent findings indicate that the rostral ACC (rACC) could receive nociception from the medial thalamus and cortex to participate in the formation and modulation of pain-related negative emotions [34]. The extent to which the rACC receives DAergic fiber projections from the VTA and the role of neurons expressing DA receptors D1 and D2 in pain modulation is yet to be fully understood.

Traditional methods, such as electrochemical stimulation and ablation, activate or inhibit various neuron types non-specifically and are inadequate for isolating the effects of DAergic neurons and their downstream regions containing DA receptor-expressing neurons. Consequently, this study utilized chemogenetic and optogenetic techniques to selectively control the excitation or inhibition of specific neuron classes, facilitating the investigation of their role in pain modulation. We developed an animal model of stress-induced CPP and employed behavioral testing, electrophysiology, chemogenetics, and optogenetics to analyze the regulatory mechanisms of the DA pathway. This approach elucidated the DA pathway's role in stress-induced CPP, examining both upstream and downstream regions of the VTA-rACC neural circuit. These findings provide a new theoretical foundation and strategic target for treating CPP.

## Materials and methods

### Animals

C57BL/6 wild-type male mice, aged 8–10 weeks and weighing approximately 25 g, were obtained from Zhengzhou University's Experimental Animal Center. DAT-cre mice were acquired from GemPharmatech LLC. The mice were maintained in cages at a controlled temperature (24 ± 2 °C) and humidity (50–60%), under a 12 h/12 h light/dark cycle. They had unrestricted access to chow and filtered water. Groups of C57BL/6 wild-type (n = 12–16 mice/group) and DAT-cre (n = 10–12 mice/group) male mice were randomly assigned to control and experimental groups. All procedures were designed to minimize animal suffering and reduce the number of animals used, while ensuring statistical accuracy. No analgesia was administered after surgery since the incision in the mice was small and recovered quickly. Experimental protocols conformed to ARRIVE guidelines, adhered to the National Institutes of Health Guide for the Care and Use of Laboratory Animals, and were approved by the Ethical Committee for Animal Research of Zheng Zhou University.

### Experimental design

Experiment 1: To assess the effect of preoperative CSDS on postsurgical nociception in mice. C57BL/6 wild-type male mice (n = 12–16 mice/group) were divided into four groups: Control (no CSDS procedure or plantar incision, n = 12), Incision (plantar incision only, n = 12), Stress (CSDS procedure only, n = 16), and Stress + Incision (CSDS procedure followed by plantar incision, n = 16). The control group was anesthetized for the same duration as surgical groups to isolate the effects of anesthesia on nociception. Mechanical nociception using von-Frey filaments was measured before and after the CSDS procedure and plantar incision. Some mice underwent immunofluorescence (D1, D2, TH, c-Fos) and Western blot (D1, D2) analyses 15 days post-incision, some mice underwent immunofluorescence (TH, c-Fos) analyses 3 days post-incision.

Experiment 2: To examine how DAergic neurons affect CPP in mice. DAT-cre male mice (n = 10–12 mice/group) were divided into three groups: Control (no CSDS procedure or plantar incision, n = 10), SI-mCherry (CSDS procedure, plantar incision, and AAV5-hSyn-DIO-mCherry, n = 12), and SI-hM3Dq (CSDS procedure, plantar incision, and AAV5-hSyn-DIO-hM3Dq-mCherry, n = 12). Similar to Experiment 1, the control group was anesthetized but did not undergo surgery to isolate the effects of anesthesia. Paw withdrawal thresholds (PWTs) were assessed pre- and post-CSDS procedure and plantar incision. CNO was administered intraperitoneally starting 11 days post-incision and lasted for five days. Anxiety-like behaviors (OFT, EPM) were evaluated 15 days post-incision. All mice were then subjected to immunofluorescence analysis.

Experiment 3: To ascertain the roles of DA receptors in the rACC in CPP mice. DAT-cre male mice (n = 10–12 mice/group) were randomly allocated to five groups: Control (no CSDS procedure or plantar incision, n = 10), SI-EYFP (CSDS procedure followed by plantar incision and AAV5-hSyn-DIO-EYFP, n = 12), SI-ChR2 (CSDS procedure followed by plantar incision and AAV5-hSyn-DIO-hChR2-EYFP, n = 12), CON336 + SI-ChR2 (CSDS procedure, plantar incision, AAV9-CON336, and AAV5-hSyn-DIO-hChR2-EYFP, n = 10), and D1-shRNA + SI-ChR2 (CSDS procedure, plantar incision, AAV9-Drd1, and AAV5-hSyn-DIO-hChR2-EYFP, n = 10). The control group was anesthetized for the same duration without incision to control for anesthesia effects on nociception. PWTs were measured before and after the CSDS procedure and plantar incision. Blue light stimulation at 10 mW and 20 Hz commenced on the 11th day post-incision and continued daily. Subsequently, the mice were dissected for immunofluorescence.

### Animal model of CPP

**Fig. 1:**
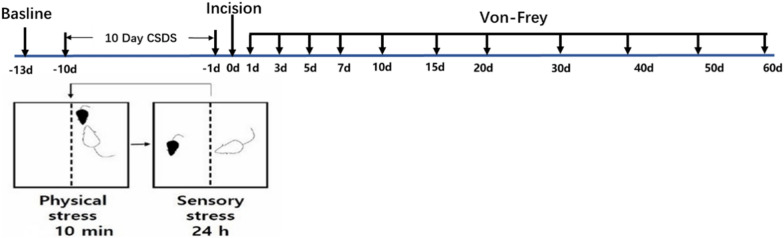
Schematic representation of an animal model of CPP

The standard CSDS protocol in mice was followed as previously described [15, 35]. CD1 mice were utilized to induce defeat in C57BL/6 J mice for 10 min daily, followed by housing both mice in a cage divided by a transparent glass partition to enhance the CD1 mice's defeat effect on the C57BL/6 J mice. This defeat regimen continued for 10 days. Refer to Fig. 1 below.

**Fig. 2:**
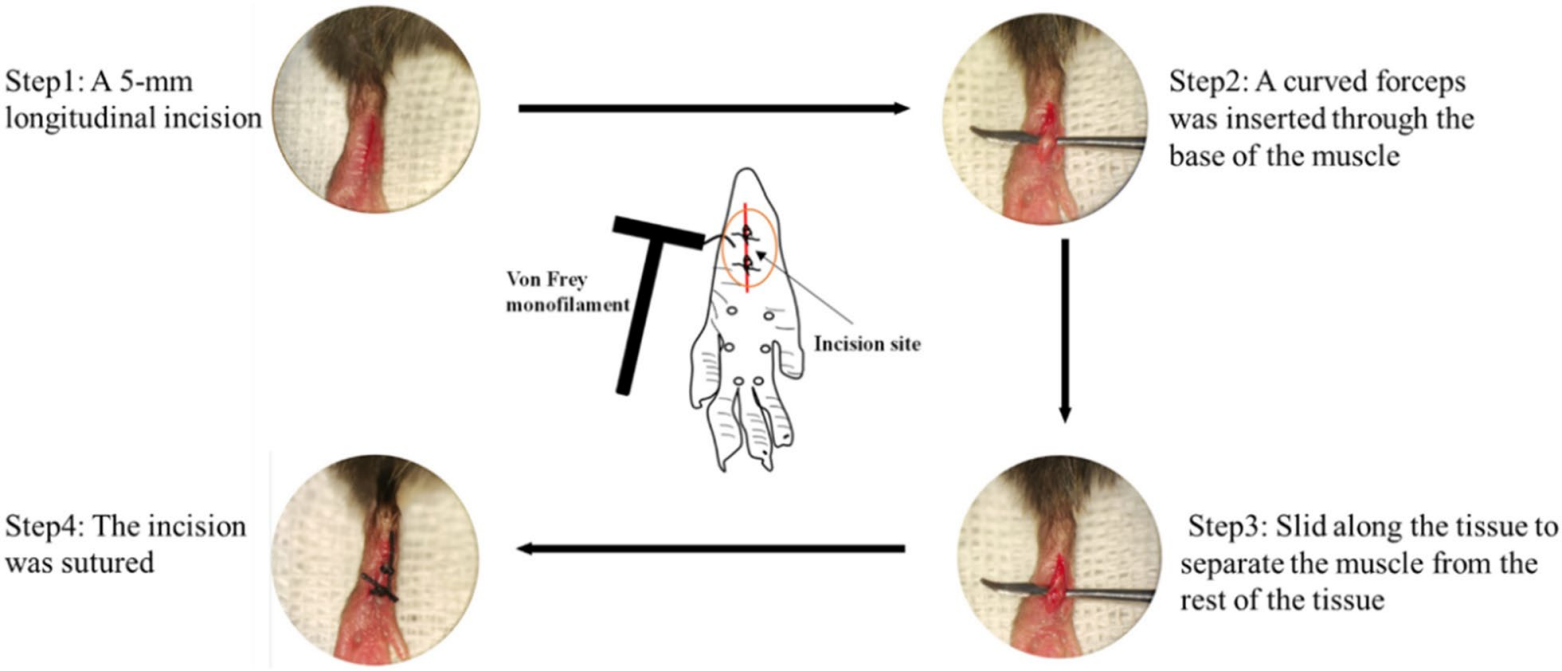
Photographs and method of plantar incision

Plantar incision surgery was performed according to previously established methods [36]. Following anesthesia, the plantar skin was cleaned with iodine. A 5-mm longitudinal incision was made in the skin, and curved forceps were inserted through the muscle base, gently separating the muscle from surrounding tissues while keeping it intact. The incision was closed with No. 1 suture, and the stitches were removed 48 h post-incision. Refer to Fig. 2 below.

### Paw withdrawal thresholds (PWTs)

Prior to testing, all mice underwent acclimatization in the test box for three days (1 h/d) to mitigate environmental influences on test outcomes. The PWTs to mechanical stimuli were assessed using calibrated von Frey filaments (Danmic/USA). Each filament was gently pressed against the plantar side of the hind paw for 1 s in ascending order, starting with a force of 0.07 g (0.07, 0.16, 0.4, 0.6, 1.0, 1.4, and 2.0 g). Baseline PWTs were measured three days before model construction and then 1, 3, 5, 7, 10, 15, 20, 30, 40, 50, and 60 days post-incision. PWTs of the mice's plantar were determined by a researcher who was blind to group assignments. Mice designated for histological analysis discontinued PWT testing 15 days after the plantar incision.

### Open field test (OFT)

The OFT was performed according to the previously detailed protocol [37]. The apparatus was a 42 × 42 × 42 cm polyvinyl chloride box. Mice were carefully placed inside, and their exploration was recorded with a video camera, analyzed using SMART3.0 (RWD) software. Each mouse was observed for 10 min. After each session, the box was cleaned with a 75% ethanol solution to remove any residual mouse scent.

### Elevated plus maze (EPM)

The EPM test was conducted following the established protocol [38]. Comprising polyvinyl chloride, the maze included a central zone (10 × 10 cm), two open arms (50 × 10 cm), and two closed arms (50 × 10 cm). Mice were placed gently in the central zone facing a closed arm, and their movements were monitored for 5 min using recording software. The apparatus was cleaned with a 75% ethanol solution after each test to avoid scent residue from previous mice.

### Methods of tissue sampling and euthanasia in mice

Following behavioral tests, mice were anesthetized with an intraperitoneal injection of urethane (1.5 g/kg). Once anesthetized, the mice were immediately secured on the operating table for thoracotomy. The left ventricle was sequentially perfused with 0.9% saline and 4% paraformaldehyde. Subsequently, the VTA and rACC regions of mice were promptly extracted by dissection for immunofluorescence staining.

### Immunofluorescence staining

The excised brain tissue was immersed in 30% sucrose solution and stored in a refrigerator until it sank. It was then mounted on a freezing microtome, with the slice thickness set at 20 μm. Following consecutive sectioning, each slice was blocked with 200 μl of 5% donkey serum for 40 min at room temperature for blocking. The primary antibodies (TH, 1:500, Abcam, ab6211; c-Fos, 1:1000, Abcam, ab208942; D1, 1:200, Abcam, ab279713; D2, 1:200, Abcam, ab313857) were then applied and incubated overnight at 4 °C. Fluorescent secondary antibodies (goat anti-mouse 647,1:1000, Abcam, ab150115; goat anti-mouse 488, 1:1000, Abcam, ab150117; goat anti-rabbit 647, 1:1000, Abcam, ab150083; goat anti-mouse 488, 1:1000, Abcam, ab150081) were added and incubated for 2 h at room temperature away from light. The sections were then mounted on slides, covered with DAPI (Abcam, ab104139) anti-fade solution, and observed under a fluorescence microscope. Three slices per animal were analyzed. The number of fluorescent neurons in each VTA or rACC slice was counted at 40X magnification, and the average value from the three slices was calculated for statistical analysis.

### Stereotactic injection in the brain

Animals were anesthetized using isoflurane in an inhalational anaesthesia device (5% for induction and 1–2% for maintenance through the nose using a face mask) and monitored for proper anaesthesia throughout the procedure by testing for the absence of the pedal reflex. The mice were placed on the stereotaxic frame with a heat-controlled pad maintained at about 35 °C and fixated with ear bars. Following thorough disinfection, a small incision was made in the cranial roof skin, exposing the bregma and lambda landmarks for precise calibration of the cranial plane. After setting coordinates, a 0.4 mm diameter drill bit was used to bore into the skull. Microsyringes then administered AAV5-EF1a-DIO-hChR2-EYFP or AAV5-hSyn-DIO-hM3Dq-mCherry (5 µl, Hamilton, NV, USA) bilaterally into the VTA (coordinate: 4.0 mm after bregma, 0.4 mm off left and right side, depth 4.5 mm). In the rACC (coordinates: 1.6 mm anterior to bregma, 0.4 mm lateral to the right side, depth 2.4 mm), D1-shRNA (AAV9-Drd1, 49750-12) or CON336 (AAV9-CON336) were injected over approximately 10 min, with the needle remaining in place for a similar duration. After completing all injections, the skull and skin were cleaned and sutured. The mice were then placed under a heating lamp until they recovered from anesthesia, became fully mobile and alert.

### Optical fibre embedding in the brain

Three days following a left posterior plantar incision, the mice were anesthetized with isoflurane (5% for induction and 1–2% for maintenance) and secured to the stereotaxic instrument. Full exposure of bregma and lambda allowed for accurate alignment with the calvarium plane. The rACC virus injection site (coordinates: 1.6 mm anterior to bregma, 0.4 mm left and right to bregma, depth of 2.2 mm) served as a reference point. An optical fiber with a ceramic head was precisely positioned in the rACC, approximately 200 nm above the virus injection site, and secured to the skull with dental cement. The mice were returned to their cages after the cement dried and they regained consciousness. One week after implantation, blue light stimulation was initiated, and mechanical PWTs were assessed.

### Quantitative real‐time PCR (qRT − PCR)

Total RNA was extracted using a RNeasy kit (Invitrogen) according to the manufacturer’s instructions. Complementary DNA (cDNA) was synthesized using a SuperScript™ III First‐Strand Synthesis System (Invitrogen Thermo Fisher Scientific, 18 080 051). qRT–PCR was performed with GoTaq® qPCR Master Mix (Promega, A6002) following the manufacturer’s protocol and a LightCycler 480 Real‐Time PCR System (Roche). The thermal cycling conditions were as follows: hold stage, 95 °C for 10 min; PCR stage, 40 cycles of 95 °C for 10 s, 60 °C for 10 s, and 72 °C for 30 s. To analyze gene expression in mouse treated with shRNA, gene-specific primers for D1 (5′‐ACCTGTCCTGGTACGATAGTG‐3′ (forward), 5′‐GCATGGCATAGTAGTTGTAGTGG‐3′ (reverse)) and GAPDH (as an internal control) were used (Integrated DNA Technologies). Each sample was run in triplicate and the average Ct value used. The RNA levels of the target gene D1 were normalized against those of GAPDH using the ΔΔCt method.

### Western blot

Mice were anaesthetized with 2% isoflurane, followed by rapid decapitation for rACC extraction. Tissue was pulverized using an ultrasonic cell disintegrator in ice-cold RIPA buffer, supplemented with a proteinase inhibitor mixture. Subsequently, samples were homogenized and centrifuged at 12,000 × g for 10 min at 4 °C. Supernatants were retrieved, and protein concentrations determined with a BCA protein assay kit. Proteins, after denaturation, underwent SDS‒PAGE and were transferred onto PVDF membranes. Membranes were blocked with 5% BSA in TBST and then trimmed based on the marker to remove non-relevant sections. These membranes were then incubated with various primary antibodies (D1, 1:200, Abcam, ab279713; D2, 1:200, Abcam, ab313857; β-actin, 1:10000, ABclonal, AC026) overnight at 4 °C. Following incubation with appropriate HRP-conjugated secondary antibodies (goat anti-rabbit, 1:5000, Abcam, ab6721), membranes were visualized using ECL-Plus detection kit (GE Healthcare Biosciences, USA).

### Statistical analysis

Group sizes were determined based on a power analysis (G*Power 3.1.9.7), estimating the necessary sample size-given α, power, and effect size [39]. Two independent sample mean differences were analyzed using t-test. Preliminary parameters were: two tails, effect size δ = 1.8, α error prob = 0.05, power 1—β = 0.8, allocation ratio *N*2/*N*1 = 1, yielding an optimal group size of 6. Consequently, 6 animals were utilized for Western blot or immunohistochemistry and 10–12 for behavioral experiments. Multiple sample differences were evaluated by F-test and ANOVA (fixed effects, omnibus, one-way), with preliminary parameters: two tails, effect size δ = 0.8, α error prob = 0.05, power 1—β = 0.8, number of groups = 3 or 4, Nonsphericity correction = 1). The optimal group size was 6 or 7, leading to the use of 6 animals for western blot or immunofluorescence and 12–16 for behavioural experiments.

Data analysis and graph generation were executed using GraphPad Prism 8.0. The Anderson–Darling normality test and Levene's Test for homogeneity of variances confirmed the validity of the analysis. Comparisons between two groups were conducted using t-test. PWTs in each group were compared using two-way RM ANOVA followed by Bonferroni's post-hoc test. Other experimental data were analyzed using one-way ANOVA and Bonferroni's post-hoc test. All results are expressed as mean ± S.E.M, and *P* < 0.05 was considered statistically significant.

## Results

### CSDS promoted chronicity of postsurgical nociception in male mice

**Fig. 3:**
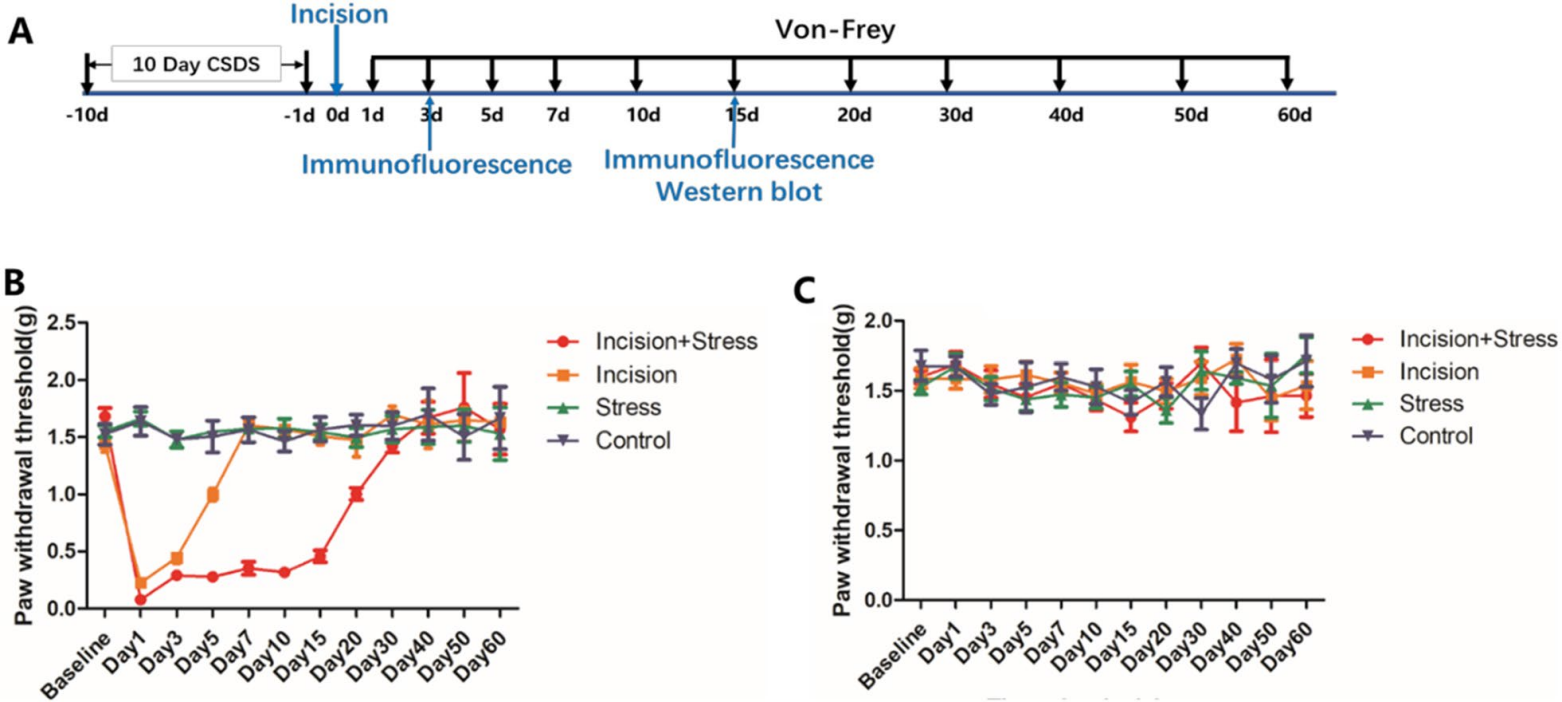
CSDS promoted chronicity of postsurgical nociception in male mice. **A** Experimental procedure schematic; **B** Left plantar PWTs (Control, n = 12; Incision, n = 12; Stress, n = 16, Stress + Incision, n = 16, Two-way RM ANOVA, interaction: F_(33;572)_ = 53.33, *P* < 0.001, Time: F_(11;572)_ = 121.3, *P* < 0.001, Group: F_(3;572)_ = 80.17, *P* < 0.001).; **C**. Right plantar PWTs (Control, n = 12; Incision, n = 12; Stress, n = 16, Stress + Incision, n = 16, Two-way RM ANOVA, interaction: F_(33;572)_ =  = 1.248, *P* > 0.05, Time: F_(11;572)_ = 0.7852, *P* > 0.05, Group: F_(3;572)_ = 0.0902, *P* > 0.05). Data are represented as mean ± SEM, Data are represented as mean ± SEM, compared with the control group, *P < 0.05, ***P < 0.001; compared with the incision group, #*P* < 0.05, ##*P* < 0.01, ###*P* < 0.001

Behavioural responses to mechanical stimulation were observed at various intervals post-incision (Fig. 3A). Results indicated a significant decrease in PWTs on the left plantar post-incision. Compared to the control group, PWTs in the incision group returned to baseline within approximately 7 days. However, the Stress + Incision group, which underwent CSDS prior to incision, displayed persistently low PWTs for at least 30 days post-incision. Stress alone did not affect PWTs (Fig. 3B). No significant changes in PWTs were observed in the right plantar across all groups (Fig. 3C). This established a viable mouse model for CPP induced by CSDS.

### Decreased activity of DAergic neurons in CPP mice

**Fig. 4:**
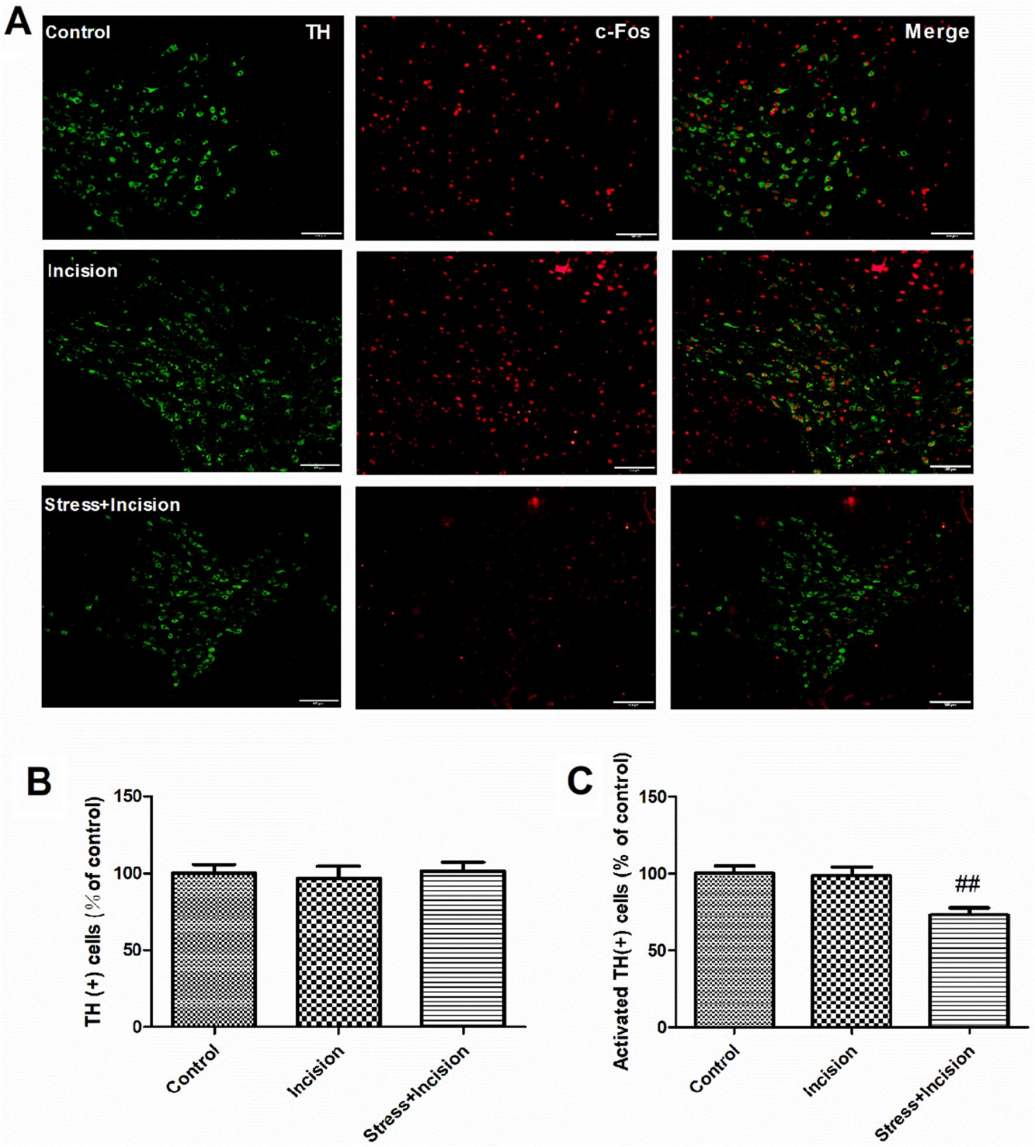
Decreased activity of DAergic neurons in CPP mice. **A** Immunofluorescence staining for TH- and c-Fos-labelled positive cells in VTA of each group, Tyrosine Hydroxylase (TH) in green, c-Fos in red; scale bars = 100 μm. **B** Quantitative analysis of TH-positive cells (One-way ANOVA, F_(2,15)_ = 0.04807, *P* = 0.9532, n = 6 for each group). **C** Quantification of cells double-labeled for TH and c-Fos (One-way ANOVA, F_(2,15)_ = 9.390, *P* = 0.0023, n = 6 for each group). Data are represented as mean ± SEM, compared with the control group; ##*P* < 0.01

DAergic neurons activation in the VTA was evaluated through immunofluorescence colocalization of TH and c-Fos, 15 days post-plantar incision. Analysis indicated no significant differences in TH-positive neuron counts in the VTA among groups (Fig. 4A, B). While the incision group exhibited no significant change in TH-positive neurons expressing c-Fos compared to the control, the Stress + Incision group demonstrated a significant reduction (Fig. 4A, C). This suggests a reduction in DAergic neuron activity in the VTA 15 days after plantar incision in mice subjected to CSDS. Further analysis revealed an increase in TH-positive neurons expressing c-Fos in the incision group 3 days post-incision compared to the control, indicating early-stage activation of DAergic neurons in incision nociception. The expression of c-Fos in DAergic neurons was significantly lower in the stress group than in the control (Additional file 1: Fig. S1), highlighting the impact of CSDS on DAergic neuron activation in the VTA, particularly during the early stages of incision-induced nociception.

### Excitation of DAergic neurons alleviates nociceptive responses and anxiety-like behaviors

**Fig. 5:**
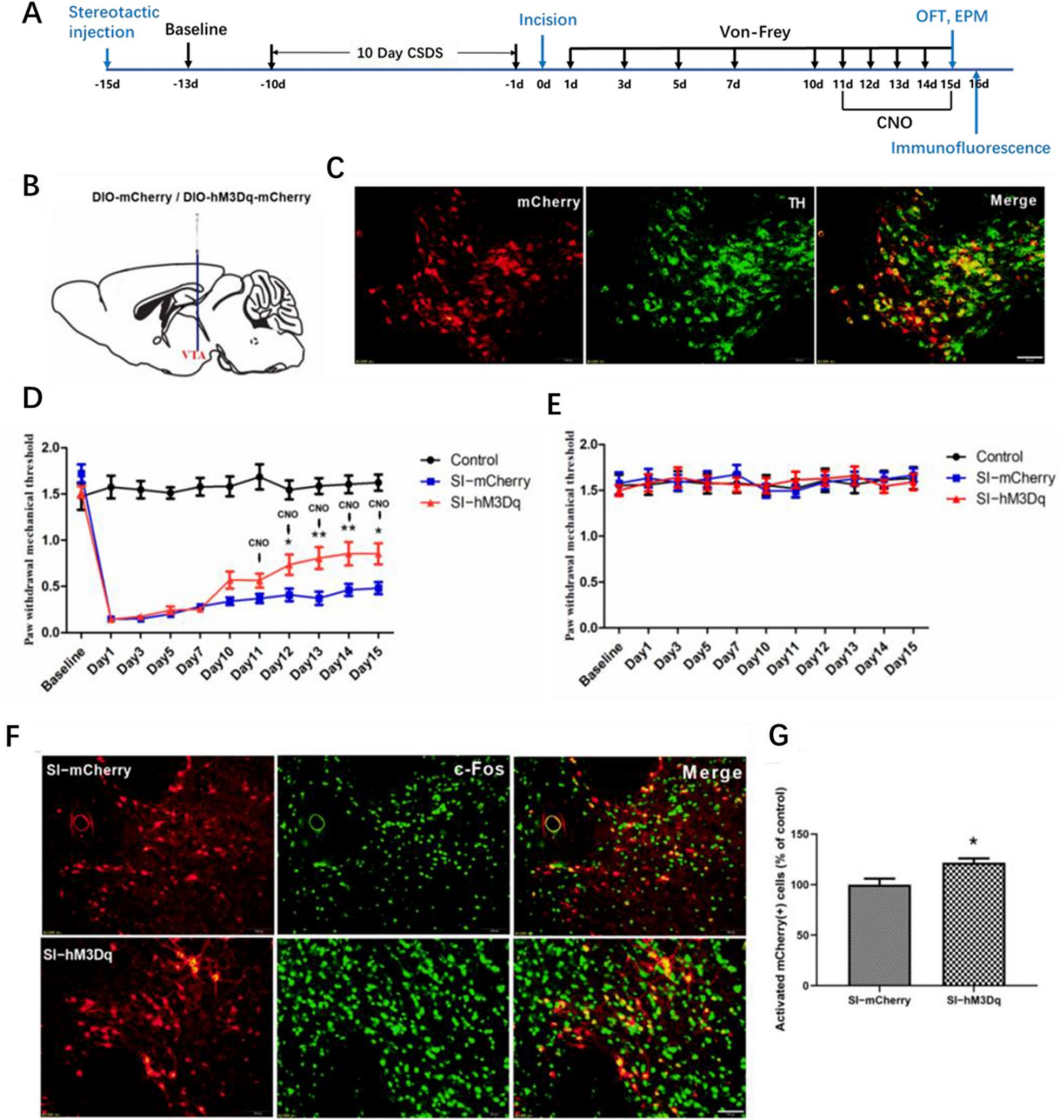
Alleviation of Nociceptive Responses by Excitation of DAergic Neurons. **A** Experimental procedure schematic; Open field test (OFT) and elevated plus maze (EPM) experiment were performed at 15 days after incision. **B** Brain virus injection in the ventral tegmental area (VTA); **C** Fluorescence staining showing virus infection and co-labeled TH-positive neurons; scale bars = 100 μm. **D** Left plantar PWTs in each group (Control, n = 10; SI-mCherry, n = 12; SI-hM3Dq, n = 12, Two-way RM ANOVA, interaction: F_(20;310)_ = 22.91, *P* < 0.001, Time: F_(10;310)_ = 76.12, *P* < 0.001, Group: F_(2;310)_ = 224.7, *P* < 0.001). **E** Right plantar PWTs in each group (Control, n = 10; SI-mCherry, n = 12; SI-hM3Dq, n = 12, Two-way RM ANOVA, interaction: F_(20;310)_ = 0.9955, *P* > 0.05, Time: F_(10;310)_ = 1.429, *P* > 0.05, Group: F_(2;310)_ = 0.0614, *P* > 0.05).** F** Immunofluorescence staining of mCherry and c-Fos labelled positive cells in each group; scale bars = 100 μm. **G** Percentage of mCherry and c-Fos double-labelled cells among the virus-infected positive cells (SI-mCherry, n = 6; SI-hM3Dq, n = 6, unpaired t-test, *P* = 0.0203). Data are represented as mean ± SEM, compared with the SI-mCherry group: *P < 0.05, **P < 0.01

The findings indicate a reduction in DAergic neuron activity in CPP mice. Consequently, this experiment aimed to investigate the influence of excitatory DAergic neurons on CPP. Clozapine oxide (CNO) was administered intraperitoneally to activate hM3Dq-expressing neurons in DAT-cre mice (Fig. 5A, B). Significant mCherry expression in DAergic neurons was observed (Fig. 5C). Post-plantar incision, CNO was administered intraperitoneally for 5 days starting from day 10, with PWTs recorded 1 h after each CNO injection. PWTs in the Stress + Incision (SI) mice of the SI-hM3Dq group showed a substantial increase from day 12, remaining higher than those in the SI-mCherry group on day 15 post-incision (Fig. 5D). No significant PWT variations were observed in the right plantar of any group (Fig. 5E).

Immunofluorescence confirmed the activation of hM3Dq-positive neurons in the VTA. Compared to the SI-mCherry group, a significant increase in hM3Dq-positive neurons expressing c-Fos was observed in the VTA of the SI-hM3Dq group (Fig. 5F, G). These findings indicate that targeted stimulation of DAergic neurons in the VTA can effectively alleviate mechanical nociception sensitivity in CPP mice.

**Fig. 6:**
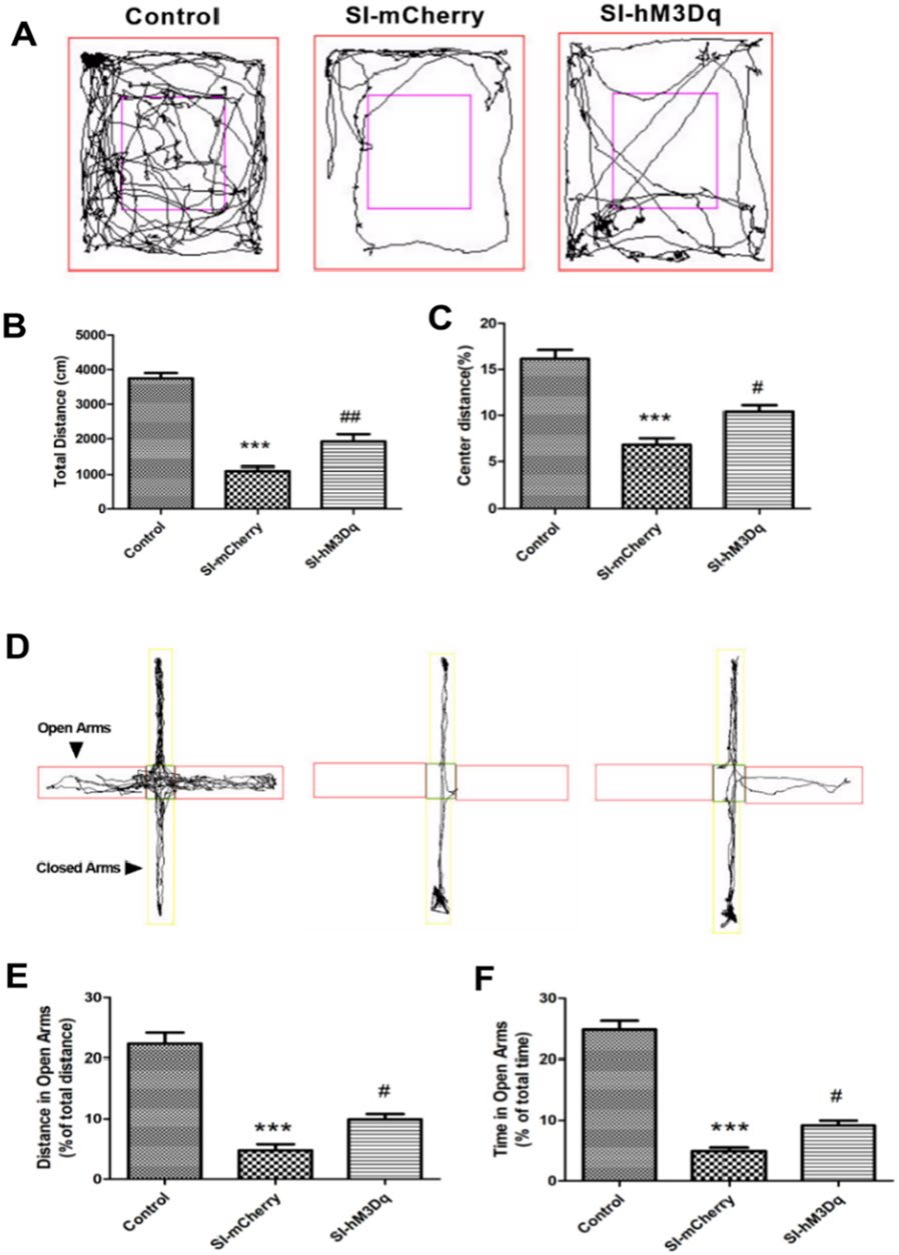
Excitation of DAergic neurons alleviates anxiety-like behaviour in CPP mice. **A** Movement tracks for each mouse group in the OFT 15 days post-plantar incision. **B** Total distance traveled by each group 15 days post-plantar incision (Control, n = 10; SI-mCherry, n = 12; SI-hM3Dq, n = 12, one-way ANOVA, F_(2,31)_ = 183.4, *P* < 0.0001). **C** Central area distance covered by each group 15 days post-plantar incision (Control, n = 10; SI-mCherry, n = 12; SI-hM3Dq, n = 12, one-way ANOVA, F_(2,31)_ = 23.05, *P* < 0.001). **D** Movement track diagrams for each group in the EPM 15 days post-plantar incision. **E**. Distance statistics in the open arm 15 days post-plantar incision for each group (Control, n = 10; SI-mCherry, n = 12; SI-hM3Dq, n = 12, one-way ANOVA, F_(2,31)_ = 77.58, *P* < 0.001). **F** Time statistics in the open arm 15 days post-plantar incision for each group (Control, n = 10; SI-mCherry, n = 12; SI-hM3Dq, n = 12, one-way ANOVA, F_(2,31)_ = 70.86, *p* < 0.001). Data are represented as mean ± SEM, compared with the control group, ***P < 0.001; compared with the SI-mCherry group, #*P* < 0.05, ##*P* < 0.01

Figure 6A depicts the activity track of mice in the OFT over 5 min. OFT results revealed increased total distance and percent of distance traveled in central areas by the SI-hM3Dq group post-CNO treatment, compared to the SI-mCherry group (Fig. 6B, C). Figure 6D illustrates the activity track of mice in the EPM for 5 min. EPM results indicated that mice in the SI-hM3Dq group showed an increased percent of distance traveled and time spent in open arms post-CNO treatment, compared to the SI-mCherry group (Fig. 6E, F).

### DAergic fibre projections of the VTA-rACC neural pathway

**Fig. 7:**
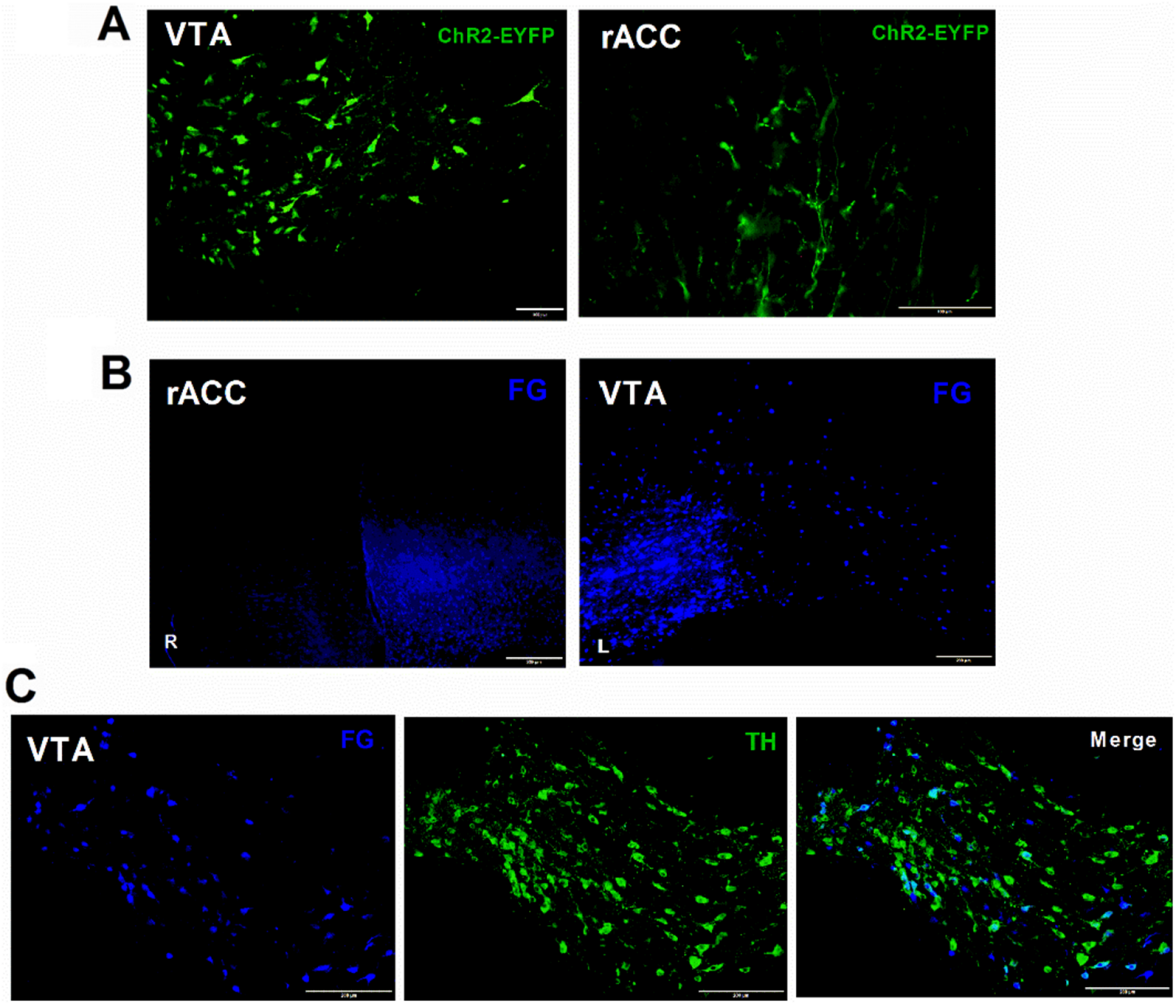
DAergic fibre projections in the neural circuit. **A** Viral expression in the ventral tegmental area (VTA) and rostral anterior cingulate cortex (rACC) regions of DAT-Cre mice. **B** FG expression in the rACC and VTA, scale bars = 200 μm. **C** Double immunofluorescence staining of FG-labelled and TH-positive neurons in the VTA; FG in blue, TH in green; scale bars = 100 μm

Four weeks post-introduction of adeno-associated virus AAV5-EF1a-DIO-hChR2-EYFP into the VTA of DAT-Cre mice, DAergic neurons and their projection fibres were marked green by EYFP. EYFP-labelled neurons in the VTA region, and EYFP-labelled filamentous or beaded projection fibers were observed in the rACC (Fig. 7A). To elucidate the VTA-rACC projection relationship, FG labelling in the rACC and FG-labelled neuron types in the VTA were observed one week after injecting retrograde tracer fluorescent gold (FG) into the rACC region of mice. The findings revealed that FG-labelled neurons in the VTA were observed following rACC injection (Fig. 7B). Double immunofluorescence staining revealed most FG-labelled neurons colocalized with TH-positive neurons (Fig. 7C), indicating that VTA-rACC projections are predominantly composed of DAergic fibres.

### Excitation of the axon terminals of DAergic neurons in the rACC alleviates nociceptive response

**Fig. 8:**
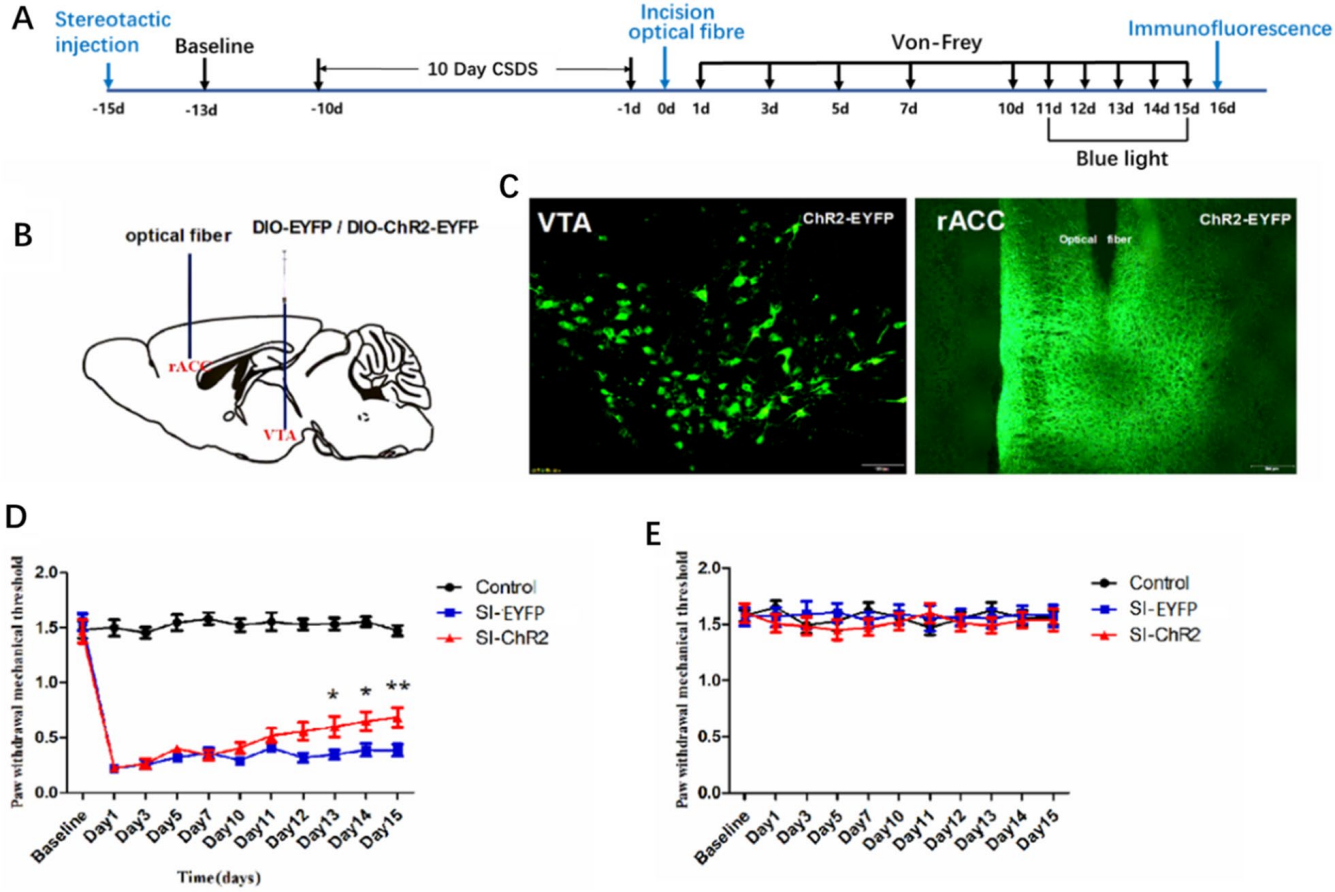
Excitation of DAergic neuron axon terminals in the rACC alleviates the nociceptive response. **A** Experimental procedure schematic. **B** Optical fibre and virus injection model of the brain. **C** Fluorescence map of virus infection in the VTA and rACC; scale bars = 100 μm. **D** PWTs of the left plantar in each group (Control, n = 10; SI-mCherry, n = 12; SI-hM3Dq, n = 12, Two-way RM ANOVA, interaction: F_(20;310)_ = 33.63, *P* < 0.001, Time: F_(10;310)_ = 111.6, *P* < 0.001, Group: F_(2;310)_ = 420.0, *P* < 0.001). **E** PWTs of the right plantar for each group (Control, n = 10; SI-mCherry, n = 12; SI-hM3Dq, n = 12, Two-way RM ANOVA, interaction: F_(20;310)_ = 1.772, *P* > 0.05, Time: F_(10;310)_ = 1.744, *P* > 0.05, Group: F_(2;310)_ = 0.1191, *P* > 0.05). Data are represented as mean ± SEM, compared with the SI-EYFP group: *P < 0.05. **P < 0.01

While we confirmed DAergic fibre projections of the VTA-rACC neural pathway, the modulation of DAergic neurons projecting from the VTA to the rACC on nociceptive response remains an area of exploration. Optical fibres were implanted in the rACC after injecting viruses into the VTA region (Fig. 8A, B). We observed EYFP-labelled neurons in the VTA and EYFP-labelled nerve fibre structures, together with the sites of optical fiber implantation in the rACC (Fig. 8C). Starting on the 10th day after plantar incision, blue light stimulation of 10 mW and 20 Hz was administered daily in the rACC, following a 2 min ON-2 min OFF-2 min ON stimulation pattern. During this stimulation, the PWTs of the mice were measured. The PWTs in the left plantar of SI-EYFP and SI-ChR2 group mice exhibited a notable decrease after plantar incision compared to the control group mice. Continuous blue light stimulation in the rACC significantly enhanced the PWTs in the left plantar of SI-ChR2 group mice, compared to the SI-EYFP group, while the right plantar PWTs in mice remained constant (Fig. 8D, E).

### Excitation of DAergic neurons alleviate the nociceptive response through D1 receptor

**Fig. 9:**
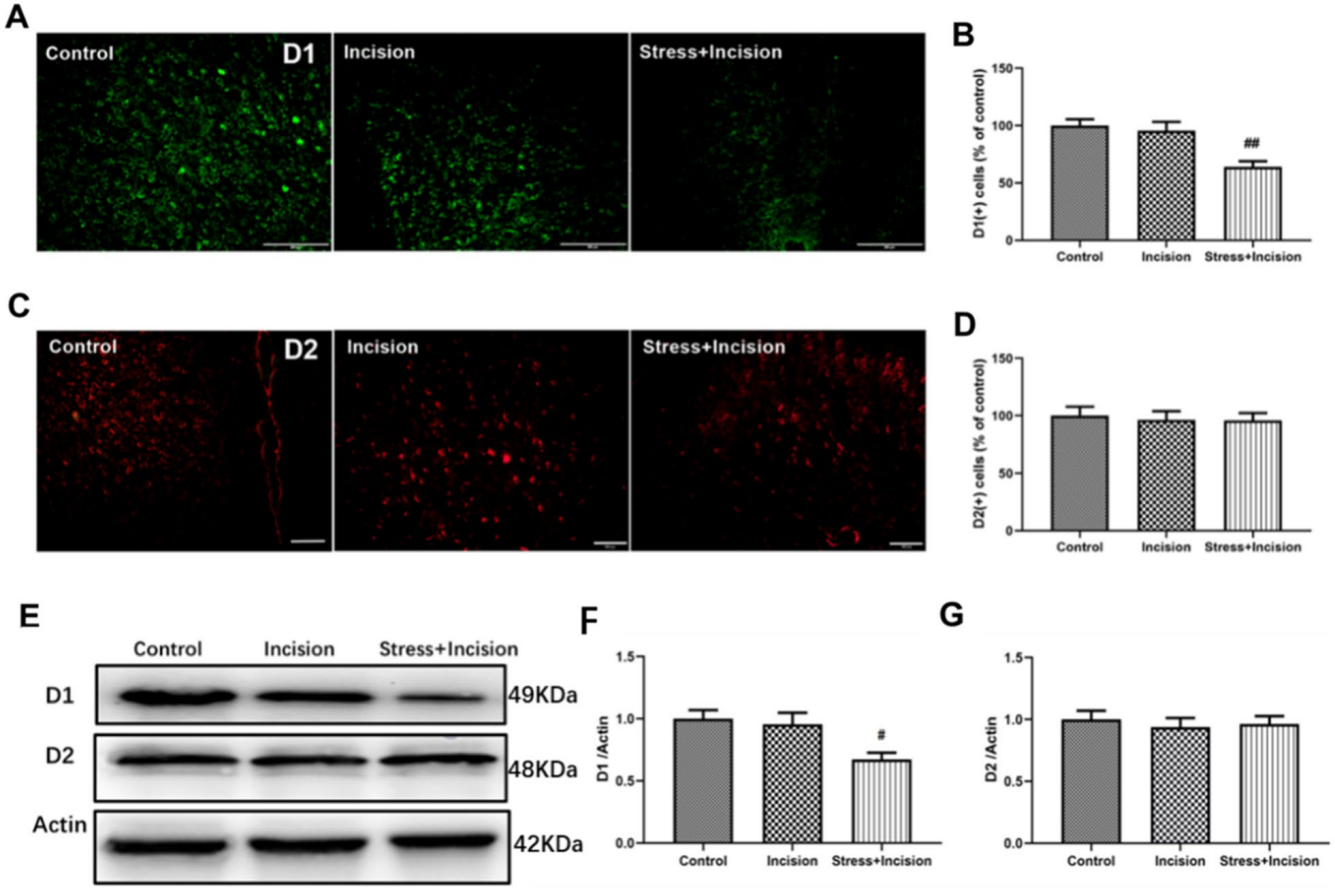
Reduced expression of DA receptor D1 in CPP mice. **A** Immunofluorescence staining of D1-positive cells in the rACC; scale bars = 100 μm. **B** Quantitative analysis of D1-positive cells (One-way ANOVA, F_(2,15)_ = 11.08, *P* = 0.0011, n = 6 for each group). **C** Immunofluorescence staining of D2-positive cells in the rACC; scale bars = 50 μm; **D** Statistical results of D2-positive cells (One-way ANOVA, F_(2,15)_ = 0.04574, *P* = 0.9554, n = 6 for each group). **E** Western blot images were cropped for clarity to display only proteins of interest. The original (cropped) images of full-length blots are in Additional file 2. **F** Quantitative results of D1 expression levels in each group of mice (One-way ANOVA, F_(2,15)_ = 5.506, *P* = 0.0161, n = 6 for each group). **G** Quantitative results of D2 expression levels in mice of each group (One-way ANOVA, F_(2,15)_ = 0.1869, *P* = 0.8314, n = 6 for each group). Data are represented as mean ± SEM, compared with the incision group, #*P* < 0.05, ##*P* < 0.01

The effects of DA receptor agonists and antagonists on chronic nociception have been established in previous studies [40, 41]. We employed immunohistochemical staining to assess the expression of DA receptors D1 and D2 in the rACC (Experiment 1). The results revealed no significant change in D1 expression in the rACC of the incision group 15 days post-plantar incision compared to the control group. However, there was a notable reduction in D1 expression in the stress + incision group compared to the incision group (Fig. 9A, B, F). No significant changes in D2 receptor expression in the rACC across the groups (Fig. 9C, D, G). Representative cropped D1 and D2 immunoreactive bands in the rACC are depicted in Fig. 9E, with the full-length blot accessible in Additional file 1. This indicates that the reduction in D1 expression in the rACC in mice is associated with CPP induced by CSDS.

**Fig. 10:**
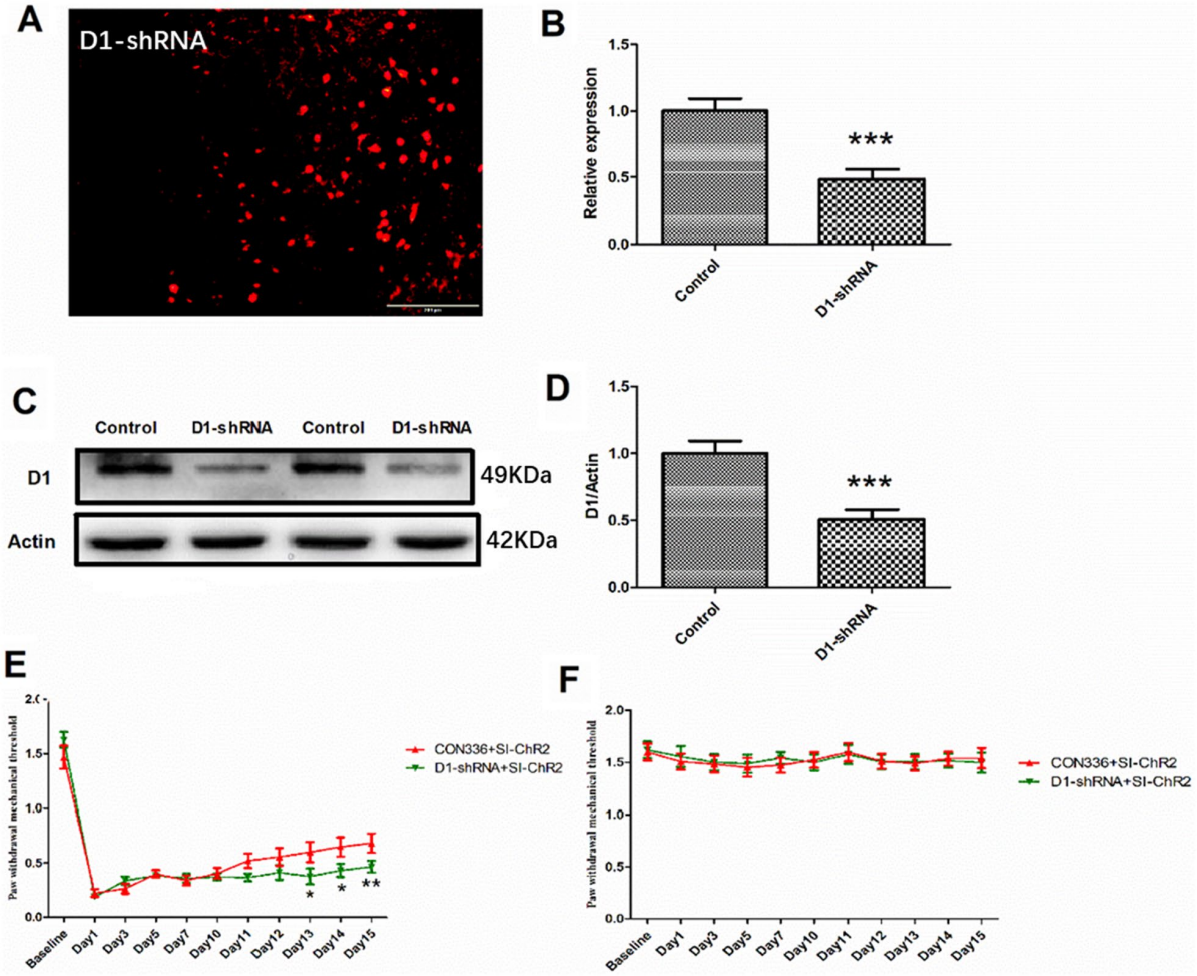
Optogenetic stimulation of DAergic neurons mitigates nociception response through D1 receptor. **A** Expression of D1-shRNA virus in the rACC; scale bars = 200 μm. **B** Statistical results the of D1 mRNA expression levels detected by qPCR (unpaired t-test, *P* = 0.0004, n = 6 for each group). **C** Western blot images were edited to exclude non-relevant sections and highlight only target proteins. Original (edited) versions of complete blots are available in Supplementary Material 2. **D** Quantitative analysis of D1 protein expression in the rACC region (unpaired t-test, *P* = 0.0006, n = 6 for each group). **E** PWTs of the left plantar in each group (Two-way RM ANOVA, interaction: F_(10;180)_ = 13.25, *P* < 0.001, Time: F_(10;180)_ = 212.0, *P* < 0.001, Group: F_(1;180)_ = 5.655, *P* < 0.05, n = 10 for each group). **F** PWTs of the right plantar in each group (Two-way RM ANOVA, interaction: F_(10;180)_ = 0.8397, *P* > 0.05, Time: F_(10;180)_ = 1.472, *P* > 0.05, Group: F_(1;180)_ = 0.1134, *P* > 0.05, n = 10 for each group). Data are represented as mean ± SEM, compared with the control group: ***P < 0.001; compared with CON336 + SI-ChR2 group: *P < 0.05, **P < 0.01

Specific activation of VTA-rACC DAergic neurons via photogenetic and chemogenetic approaches markedly alleviate mechanical nociceptive sensitivity in CPP mice, and significantly improved activity in the central area of the OFT and in the open arm of the EPM. To determine whether this analgesic effect involves the DA receptor D1 in the rACC, we examined D1 receptor expression. The findings revealed a notable reduction in D1 expression in the D1-shRNA group relative to the control group (Fig. 10A, B, C, D). Furthermore, the PWTs of the left plantar in the D1-shRNA + SI-ChR2 group were significantly lower from the 13th day post-plantar incision when compared to the CON336 + SI-ChR2 group (Fig. 10E). However, no substantial differences were observed in the PWTs of the right plantar between the two groups (Fig. 10F). These findings suggest that reduced D1 expression can counteract the PWTs increase resulting from activation of DAergic neurons.

## Discussion

In this study, we determined that DAergic neurons in the VTA project downwards to rACC neurons using morphological techniques. CSDS led to reduced activation of these neurons in the VTA. Dysfunction in the DA system might contribute to the persistence of postsurgical nociception. Targeted stimulation of DAergic neurons in the VTA can substantially reduce nociception and negative emotions, potentially through the D1 receptors in the rACC region.

CPP is commonly associated with anxiety and depression, with these negative emotions worsening pain development and recurrence. Therefore, developing an animal model that accurately simulates this psychological impact on pain is crucial. In our research, we created a mouse model that more closely mirrors the chronic progression of postsurgical pain. We observed that CSDS over 10 days extended postsurgical nociception in mice, leading to the transition from an acute to a chronic phase. This model effectively replicates CPP induced by stress. Due to the aggressive nature of male CD1 mice used in the CSDS model and their lack of aggression towards female mice, only male mice were used in this study. Changes in c-Fos protein expression in DAergic neurons in the VTA were also observed, highlighting their potential role in modulating incision nociception. Previous research has shown DAergic neurons are hypoexcitable in chronic pain states, diminishing motivation in humans and animals [42]. Our findings reveal a significant reduction in c-Fos protein expression in DAergic neurons in the VTA in mice with CSDS-induced chronic postsurgical nociception, suggesting alterations in DAergic neuron activity during chronic postsurgical nociception.

To further explore how DAergic neurons contribute to the chronicity of postsurgical nociception, we examined their activation in mice three days following plantar incision, both in the incision and stress groups (Additional file 1: Fig. S1). We discovered that CSDS causes decreased activation of DAergic neurons in the VTA. Interestingly, increased activation of these neurons was noted in the early stages of incision nociception (three days post-plantar incision), which might play a role in acute nociception recovery. This aligns with recent findings that early CCI-induced chronic neuropathic nociception increases catatonic activity and explosive discharge of DAergic neurons in the contralateral VTA region [43].

Traditional anterograde and retrograde tracers exhibit a degree of nonspecificity in fibre projection tracing, necessitating the integration of these tracers with viral techniques for improved accuracy in identifying neuronal projections [44–46]. Considerable research on the fibre projection associations of the medial prefrontal cortex (mPFC) in rodents has shown that the mPFC, particularly the PL region, receives DAergic fibre projections from the VTA [47]. In this study, fluorescently labelled projective fibres were visible in the rACC following virus injection into the VTA. To further validate the projective connection between the VTA and rACC, FG was injected into the rACC of mice, resulting in the observation of FG inversely labelled neurons in the VTA. Further analysis revealed these FG-targeted neurons colocalized with TH, signifying rACC neurons receive projections from DAergic neurons in the VTA.

Earlier studies have indicated that plantar incision does not affect the total distance mice travel in the OFT but reduces activity in its central area due to negative emotions [33]. In our study, specific activation of DAergic neurons in the VTA through optogenetics not only diminished nociceptive sensitization in mice but also significantly enhanced their central distance traveled in the OFT and the moving distance and time spent in the EPM's open arm. A photogenetic virus was administered into the VTA and optical fibers implanted into the rACC to specifically stimulate the axon terminals of DAergic neurons in the rACC. This stimulation corresponded with activation of DAergic neurons in the VTA, contributing to nociception relief. Nociception relief is perceived as a "reward" effect. Taylor et al. recently reported that disinhibition of DAergic neurons triggers DA release in the nucleus accumbens (NAc), producing a potent analgesic effect in both acute and chronic nociception [48]. Moreover, activation of DAergic neurons within the VTA is known to yield analgesic effects [49, 50]. In models of chronic neuropathic pain, DAergic neurons projecting from the VTA to the NAc are involved in nociception relief [43, 51].

Growing evidence suggests that the DA pathway plays a crucial role in the transmission and modulation of pain [52, 53]. While the impact of DA receptors on nociception has been extensively explored, the specific roles of different DA receptor subtypes remain unclear. DA receptors are categorized into two major subclasses based on pharmacological properties: D1-like (D1 and D5) and D2-like (D2, D3, and D4) receptors. In rodents' central nervous system, D1 and D2 receptors are most prevalent. To ascertain the roles of D1 and D2 receptors in the rACC in CPP, we investigated changes in these receptors' expression 15 days after a left posterior plantar incision in CSDS-exposed mice. Our findings indicate a decrease in D1 receptor expression in the rACC in the CPP mice, while D2 receptor expression remained largely unchanged. These results imply that altered D1 receptor expression in the rACC may influence DA signalling's regulatory role in CSDS-induced CPP. Given that D1 and D2 receptors are seldom co-expressed in the same cell type [54, 55], most D1 receptors are found in excitatory neurons, while D2 receptors are predominantly located in inhibitory neurons. This allows for targeted manipulation of these receptors in future research.

Elman and Borsook [56] proposed that DA levels are reduced in individuals with chronic pain, a decline potentially stemming from impaired neuronal activity in the VTA during chronic pain. Conversely, the use of DA receptor agonists has demonstrated efficacy in relieving neuropathic pain [57, 58]. In conditions like chronic pain and emotional disorders, DA neurotransmission is compromised both presynaptically and postsynaptically [24, 40, 59]. Administering nonselective DA drugs or selective DA receptor agonists into the substantia nigra or NAc can elevate the thermal nociceptive sensitivity threshold, serving an analgesic function in neuropathic pain [41, 60, 61]. In our current research, we specifically decreased D1 expression in the rACC using a viral interference technique. Following the injection of AAV9-Drd1 into the rACC, both D1 mRNA and protein levels significantly declined after 4 weeks of virus expression. Furthermore, the reduction in D1 expression counteracted the increase in PWTs resulting from the activation of DAergic neurons, suggesting that the analgesic effect of increased DA release due to DA neuron activation might be mediated by D1 receptors.

Anxiety-like behavior represents a notable behavioral manifestation in defeated mice within CSDS models. It appears that prolonged postsurgical nociception may be linked to preoperative anxiety. Yet, the connection between stress, anxiety, dopamine, and prolonged postsurgical nociception remains to be elucidated. While our research indicated that activating DAergic neurons could reduce anxiety-like behaviors in CPP mice, it did not provide conclusive evidence. Further investigation is needed to clarify the role of preoperative anxiety in dopaminergic circuits and prolonged postsurgical nociception, potentially employing anxiolytics in subsequent studies. In addition to behavioral research molecular-level analysis, including inflammatory cytokines (TNF-α, IL-1β and IL-6), should be conducted for further validation. Epidemiological studies also indicate a higher prevalence of anxiety disorders in the general population, with a higher incidence in women than in men [62]. Unfortunately, only male mice were used in our study. The effects of stress on prolonged postoperative nociception in female mice warrant future exploration.

## Conclusion

Our findings from both morphological and functional studies demonstrate that the VTA-rACC neural pathway plays a critical role in the development of CPP and the emergence of negative emotions due to CSDS. Stimulation of DAergic neurons in this pathway has been shown to yield significant analgesic effects and ameliorate negative emotions. This research offers crucial insights into the VTA-rACC pathway as a fundamental neural circuit governing the interplay between nociception and negative emotion in mouse model of CPP induced by CSDS.

### Supplementary Information

**Additional file 1: Figure S1.** Decreased activity of DAergic neurons induced by CSDS. **A** Immunofluorescence staining of TH and c-Fos labeled positive cells in the VTA region, TH in green, c-Fos in red, Scale bars = 100 μm. **B** The statistical results of TH positive cells (One-way ANOVA, F_(2,15)_=1.500, *P* = 0.2548, n = 6 for each group). **C** The percentage of TH and c-Fos double labeling in the number of TH positive cells (One-way ANOVA, F_(2,15)_=31.05, *P* < 0.0001, n = 6 for each group). Data are represented as mean±SEM, compared with the control group: *P <0.05, ***P< 0.001.

**Additional file 2**: Western blot 3 –Membrane -D1 and β-actin. The figure was prepared with the original (cropped) image obtained from the FluorChem M Imager. The PVDF membranes were cropped according to the marker to remove irrelevant sections of the membrane and incubate D1 and β-actin antibodies. The exposure time was set automatically by FluorChem M (ProtechSimple). Please see Fig.10C, D for comparison and detailed description

## Data Availability

The datasets generated and analysed during the current study are not publicly available due to avoid data leakage, but are available from the corresponding author on reasonable request.

## Abbreviations

ACC: Anterior cingulate cortex
ChR2: Channelrhodopsin-2
CPP: Chronic postsurgical pain
CSDS: Chronic social defeat stress
CNO: Clozapine-N-oxide
DA: Dopamine
EPM: Elevated plus maze
EYFP: Enhanced yellow fluorescent protein
OFT: Open field test
PWTs: PAW withdrawal thresholds
rACC: Rostral anterior cingulate cortex
VTA: Ventral tegmental area
