# Supplementary material for: Ventral tegmental area dopaminergic circuits participates in stress-induced chronic postsurgical pain in male mice
Source: BMC Neurosci. 2024 Jan 9;25:3. doi: 10.1186/s12868-023-00842-z (PMC10775611; doi:10.1186/s12868-023-00842-z)
Supplement: Supplementary file 1 — Additional file 1: Figure S1. Decreased activity of DAergic neurons induced by CSDS. A Immunofluorescence staining of TH and c-Fos labeled positive cells in the VTA region, TH in green, c-Fos in red, Scale bars = 100 μm. B The statistical results of TH positive cells (One-way ANOVA, F(2,15)=1.500, P = 0.2548, n = 6 for each group). C The percentage of TH and c-Fos double labeling in the number of TH positive cells (One-way ANOVA, F(2,15)=31.05, P < 0.0001, n = 6 for each group). Data are represented as mean±SEM, compared with the control group: *P <0.05, ***P< 0.001. [file 12868_2023_842_MOESM1_ESM.pdf]

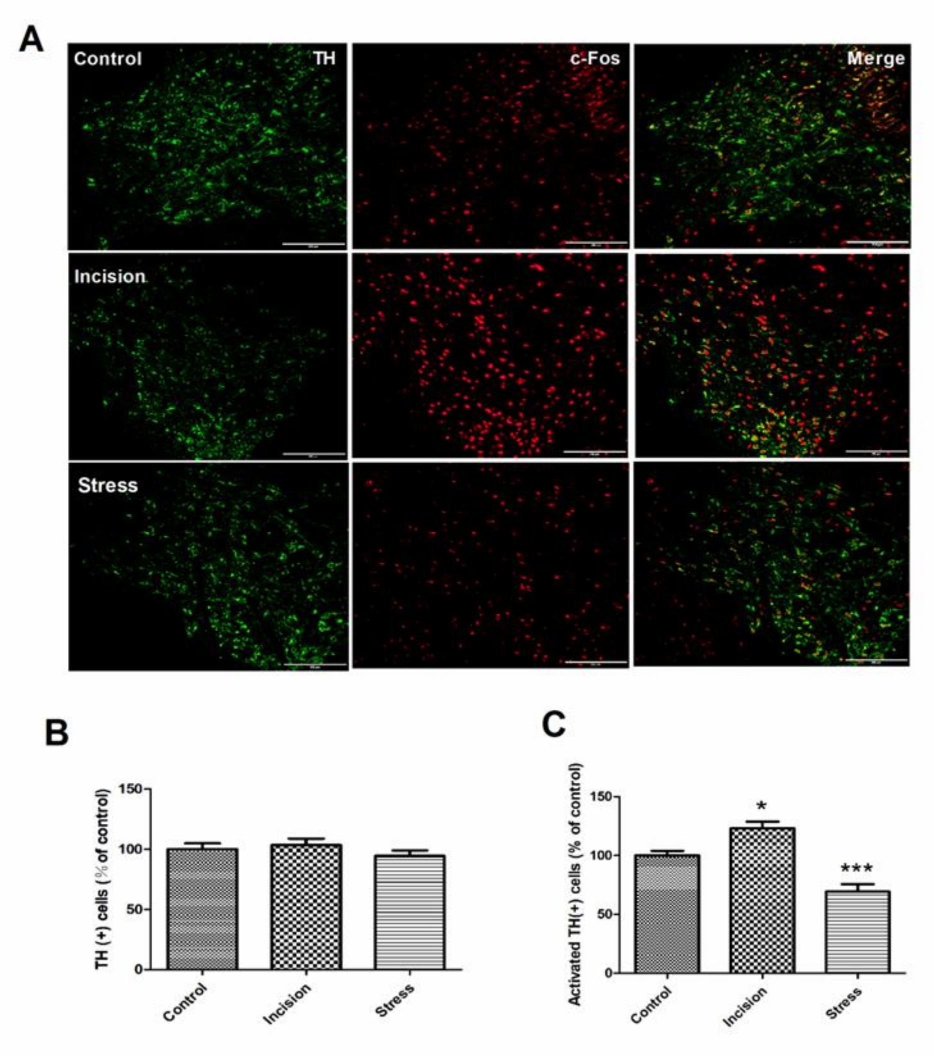

Supplementary Fig. 1. Decreased activity of DAergic neurons induced by CSDS. A. Immunofluorescence staining of TH and c-Fos labeled positive cells in the VTA region, TH in green, c-Fos in red, Scale bars = 100 μm. **B**. The statistical results of TH positive cells (One-way ANOVA, $F_{(2,15)}$=1.500, *P*=0.2548, n=6 for each group). **C**. The percentage of TH and c-Fos double labeling in the number of TH positive cells (One-way ANOVA, $F_{(2,15)}$=31.05, *P* <0.0001, n=6 for each group). Data are represented as mean ± SEM, compared with the control group: **P*<0.05, ****P*<0.001.
