# Supplementary material for: Ventral tegmental area dopaminergic circuits participates in stress-induced chronic postsurgical pain in male mice
Source: BMC Neurosci. 2024 Jan 9;25:3. doi: 10.1186/s12868-023-00842-z (PMC10775611; doi:10.1186/s12868-023-00842-z)
Supplement: Supplementary file 2 — Additional file 2: Western blot 3 –Membrane -D1 and β-actin. The figure was prepared with the original (cropped) image obtained from the FluorChem M Imager. The PVDF membranes were cropped according to the marker to remove irrelevant sections of the membrane and incubate D1 and β-actin antibodies. The exposure time was set automatically by FluorChem M (ProtechSimple). Please see Fig.10C, D for comparison and detailed description [file 12868_2023_842_MOESM2_ESM.pdf]

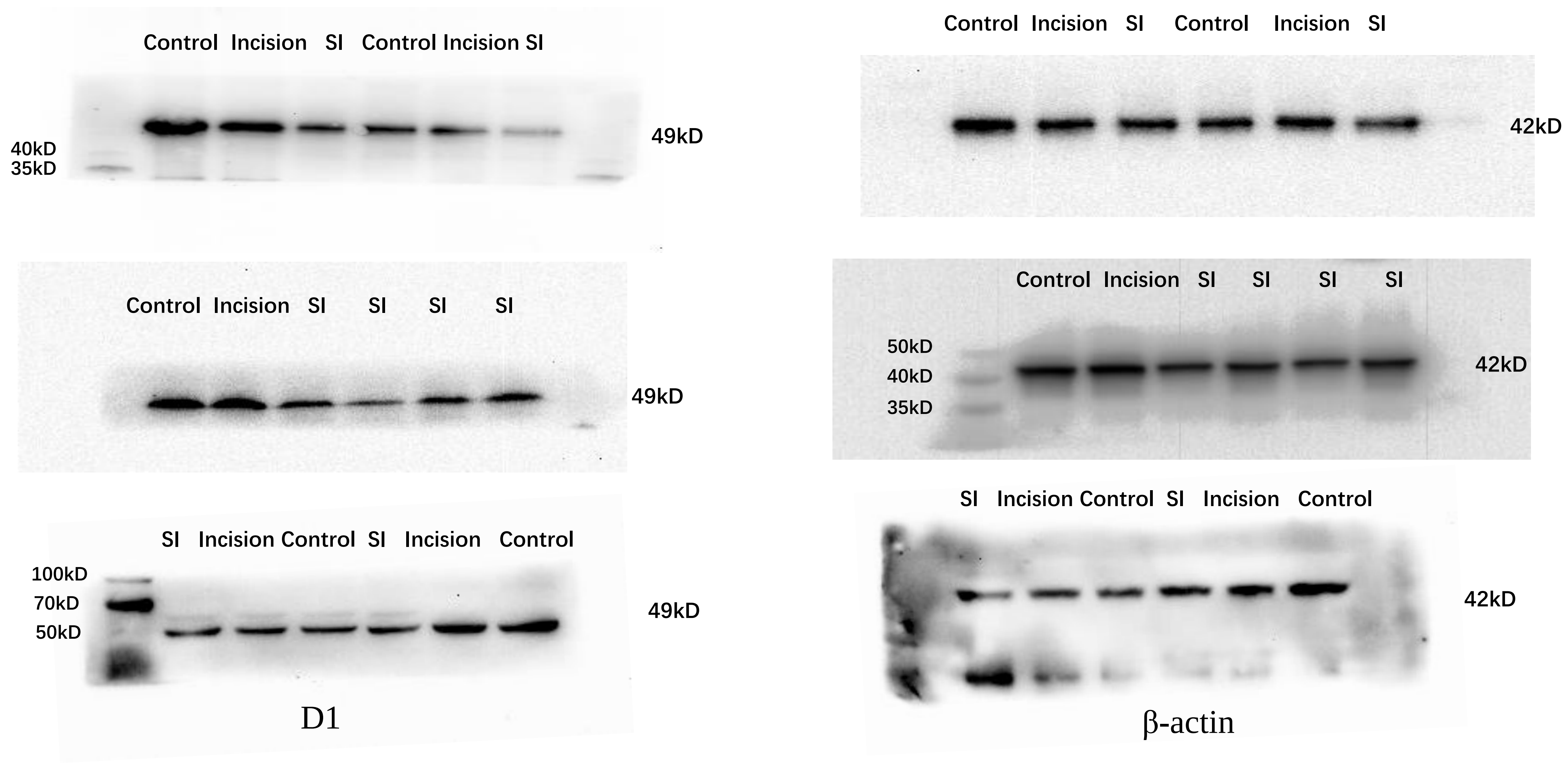

Western blot 1 – Membrane - D1 and β-actin. The figure was prepared with the original (cropped) image obtained from the FluorChem M Imager. The PVDF membranes were cropped according to the marker to remove irrelevant sections of the membrane and incubate D1 and β-actin antibodies. The exposure time was set automatically by FluorChem M (ProtechSimple). Please see Fig. 9EFG for comparison and detailed description. SI refer to Stress+Incision.

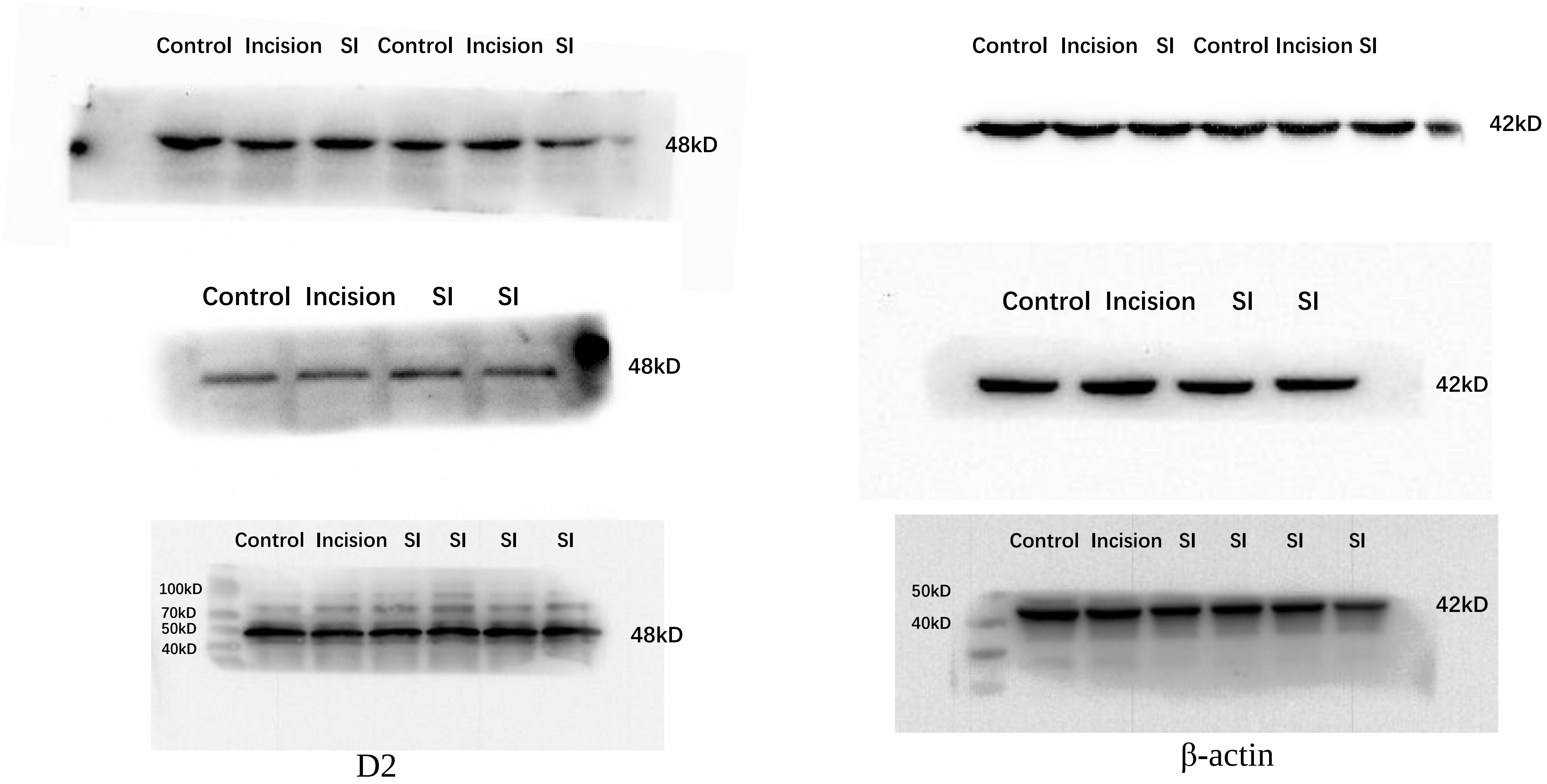

Western blot 2 – Membrane – D2 and β-actin. The figure was prepared with the original (cropped) image obtained from the FluorChem M Imager. The PVDF membranes were cropped according to the marker to remove irrelevant sections of the membrane and incubate D2 and β-actin antibodies. The exposure time was set automatically by FluorChem M (ProtechSimple). Please see Fig. 9EFG for comparison and detailed description. SI refer to Stress+Incision

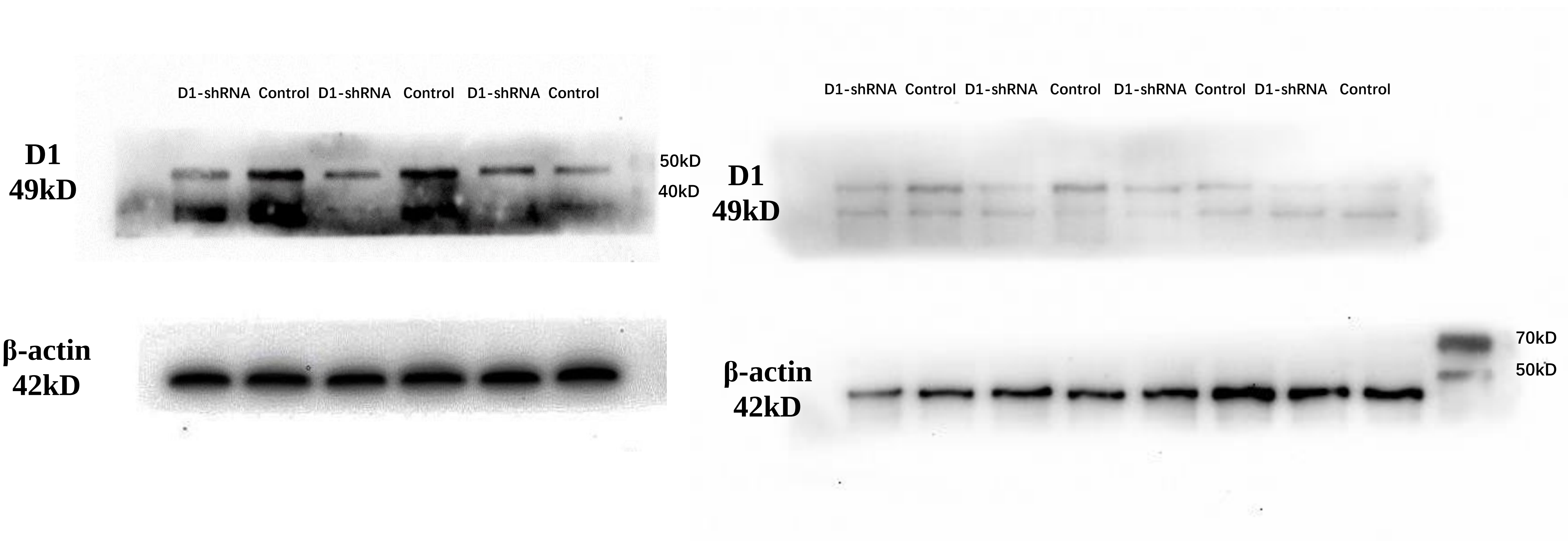

Western blot 3 – Membrane - D1 and β-actin . The figure was prepared with the original (cropped) image obtained from the FluorChem M Imager. The PVDF membranes were cropped according to the marker to remove irrelevant sections of the membrane and incubate D1 and β-actin antibodies. The exposure time was set automatically by FluorChem M (ProtechSimple). Please see Fig.10CD for comparison and detailed description
